# Depression, Loneliness and Quality of Life in Institutionalised and Non-Institutionalised Older Adults in Portugal: A Cross-Sectional Study

**DOI:** 10.3390/nursrep14030174

**Published:** 2024-09-10

**Authors:** Celso Silva, Rogério Ferreira, Bruno Morgado, Elisabete Alves, César Fonseca

**Affiliations:** 1 Higher School of Health, Polytechnic Institute of Beja, 7800-295 Beja, Portugal; 2 Instituto de Investigação e Formação Avançada, University of Evora, 7000-811 Evora, Portugal; 3 Comprehensive Health Research Centre (CHRC), University of Evora, 7000-811 Evora, Portugal; 4 Escuela de Doctorado, Universitat Rovira y Virgili, 43005 Tarragona, Spain; 5 São João de Deus School of Nursing, University of Evora, 7000-811 Evora, Portugal; 6 Nursing Department, University of Evora, 7000-811 Evora, Portugal

**Keywords:** depression, depressive symptoms, loneliness, quality of life, older adults

## Abstract

Our study aims to estimate the prevalence of depressive symptomatology among older adults and to assess their association with loneliness and quality of life according to institutionalisation status in a Portuguese sample. Background: The World Health Organisation estimates that by 2050, the world’s population over 60 will number two billion people, which poses complex challenges in terms of maintaining the mental health of older adults. The COVID-19 pandemic has increased the prevalence of depressive symptoms in this population, but the post-pandemic phase has not yet been studied much. Methods: A cross-sectional survey was carried out in 2023 among institutionalised and non-institutionalised older adults (total *n* = 525; institutionalised = 458; non-institutionalised = 67) who were selected by convenience sampling. The Patient Health Questionnaire (PHQ-9) was used to assess the presence of depressive symptoms, the WHOQOL-BREF to assess perceived quality of life and the Loneliness Scale (UCLA) to assess negative feelings of loneliness. Unconditional logistic regression models were fitted to compute crude adjusted odds ratios (ORs) and the respective 95% confidence intervals (95%CIs) for the association between sociodemographic, clinical and psychosocial characteristics and depressive symptomatology, according to institutionalisation status. The final model was adjusted for sex, age, QoL and feelings of loneliness. Results: Of the 525 participants, 74.6% of the non-institutionalised participants had no or minimal depressive symptoms, while 55.4% of the institutionalised participants fell into this category. Mild to moderately severe depressive symptoms were present in 25.4% of the non-institutionalised participants. 26.9% of the institutionalised participants had mild symptoms, 11.8% had moderate symptoms, 3.9% had moderately severe symptoms, and 2.0% had severe depressive symptoms. Overall, a higher quality of life was associated with lower levels of depressive symptoms. Participants describing feelings of loneliness were more likely also to present depressive symptoms (OR = 78.10; 95%CI 2.90–2106.08 and OR = 3.53; 95%CI 1.72–6.91 for non-institutionalised and institutionalised older adults, respectively), independently of institutionalisation status. Conclusions: The prevalence of depressive symptoms is high in older adults, which means that it has not decreased after the increase seen due to the COVID-19 pandemic. A lower perception of quality of life and the presence of negative feelings of loneliness are associated with the presence of depressive symptoms. These conclusions suggest that plans should be developed to intervene in the dimensions of depressive symptoms, perceived quality of life and negative feelings of loneliness.

## 1. Introduction

The association between depression, loneliness and quality of life is complex and multi-faceted, particularly in older adults. Research indicates that both depression and loneliness can significantly affect perceived quality of life. Depression is a prevalent problem in older adults and, if combined with loneliness, can decrease perceived quality of life [1]. Loneliness may also partially mediate the association between depression and quality of life in older adults, indicating a complex interaction of psychological and sociological factors in this population [1].

There may be a direct association between depression and feelings of loneliness, with a negative impact on perceived quality of life without the mediation of other psychological or sociological factors [2]. Loneliness is associated with depressive symptoms in older adults, where higher levels of depression correlate with increased loneliness, particularly in older populations [1,3], highlighting its negative impact on mental health during ageing [4], and so the phenomenon of ageing must also be considered in its various dimensions.

The phenomenon of ageing is the natural, progressive, dynamic and irreversible process that people go through over time, accompanying them throughout their lives, from birth to death, emerging physical, biological, social, economic, cultural, environmental, historical and psychological changes that occur in the person. This process is observed in all forms of life and is an intrinsic part of the life cycle of all organisms, including humans [5,6].

In the human context, ageing is characterised by a series of changes that can vary from person to person, but some common changes include physical changes such as changes in skin, hair, nails, vision, hearing, muscle capacity and bone density. A decline in cognitive function can also occur, where people can experience difficulties with memory, concentration and the speed at which they process information. There can be an increased susceptibility to disease, as with ageing, the immune system can become less efficient, making the body more vulnerable to infections and illnesses. Changes in the emotional dimension can also arise, as older people may face emotional challenges, such as dealing with personal losses and facing issues related to mortality. Ageing can also involve changes in social relationships, such as retirement and the establishment of new family roles [5,6,7,8].

Although ageing is inevitable, there are measures people can take to promote healthy ageing and improve their quality of life as they get older. These can include a balanced diet, regular exercise, activities that stimulate the mind and participation in a social support network. It is important to recognise that ageing can also be a time of opportunity and personal growth. Society can benefit from the contribution of older people, as they have experience, wisdom and knowledge accumulated throughout their lives [6,8]. It is, therefore, essential to respect and value people as they age and to ensure that they have access to adequate resources and care to enjoy a full and meaningful life throughout their existence.

Population ageing is a phenomenon that exists in most countries around the world and has led to very profound social changes [9]. This phenomenon associated with loneliness can have serious consequences for the mental health of older adults, even more so when we know that the COVID-19 pandemic has worsened the mental health of this population group [10,11,12].

In addition, the World Health Organisation (WHO) estimates that by 2050, the world population over 60 will number two billion people [13], which poses complex challenges in terms of maintaining the mental health of older adults, namely the emergence of depressive symptoms, and because many of them live in solitude, this can affect their perception of quality of life [14]. Depressive symptoms are often the cause of disability, especially when associated with other chronic illnesses, leading to impaired functionality. Declining functionality is a concern these days, as it leads to dependency and institutionalisation [15].

Depression is a mental disorder characterised by lasting feelings of sadness, hopelessness, negativity, lack of interest in once pleasurable activities and a persistently low mood. In terms of diagnostic criteria, these symptoms must be experienced most of the day, almost every day, for a minimum period of two weeks, although it is often observed that these symptoms can last longer [16]. This disorder can result in great suffering, disability and major limitations in the person’s day-to-day functioning [17,18], as well as being associated with cognitive dysfunctions such as dementia [19,20] and higher suicide rates compared with younger ages [21].

There are studies that report that depressive symptoms are positively associated with institutionalisation in older adults, and when compared with non-institutionalised older adults, the latter have a lower prevalence of depressive symptoms [22,23,24]. The environment in which people live plays a role in the results of studies. Depression may be more prevalent in older adults living in institutions compared with those living in residential homes [22] and in long-stay situations for older adults [23,24]. Older adults living in nursing homes have higher levels of depression than those living in extended family environments, indicating a significant association between depression and residential environments [25].

Loneliness can be defined as a subjective feeling of social isolation and by the perceived disparity between an individual’s social needs and the degree to which those social needs are met through meaningful social engagements [26,27] and is associated with negative health outcomes, including depression [28], and consequently, a reduction in the feeling of loneliness is associated with a general improvement in health [29].

Increased social interaction, reducing feelings of loneliness, can reduce the prevalence of depressive symptoms in older adults [30]. Negative feelings of loneliness contribute significantly to the onset of depressive symptoms, resulting in a decrease in perceived quality of life [31], and according to Vaz Serra (2006), loneliness may be associated with a lower perception of overall quality of life. The negative feeling of loneliness is present at all ages but is more common in older adults [14], which may anticipate depressive symptoms, but depressive symptoms are not predictive of negative feelings of loneliness [32,33].

The perception of overall quality of life is also a subjective feeling, but it is assessed by the person considering various factors such as physical health, psychological factors, social relationships, the environment, healthcare and the existence of leisure time [14,34].

Negative feelings of loneliness and depressive symptomatology interfere with quality of life in older adults, but the mechanism by which depressive symptomatology mediates the association between negative feelings of loneliness and quality of life in older adults is unclear, but it can be assumed that negative feelings of loneliness can lead to the emergence of depressive symptoms, and depressive symptoms reduce the perception of quality of life [14,35].

A study carried out in the context of the COVID-19 pandemic suggests that the possibility of leaving home/institution is a protective factor in maintaining the quality of life [36], which leads us to assume that institutionalisation can be a factor in reducing the quality of life in older adults.

Institutionalisation and socialisation play crucial roles in the mental health and quality of life of vulnerable populations, particularly older adults. Institutionalised older adults have a higher prevalence of depressive symptoms, with a significant negative correlation between depressive symptoms and physical quality of life [37,38]. Developing interventions that promote socialisation can lead to better health outcomes and a better perception of quality of life [39]. So, we might think that in the population of institutionalised older adults, it could be very useful to promote socialisation, as this can lead to improvements in various dimensions [38,40].

In fact, from a mental health prevention and promotion perspective, promoting socialisation in institutional settings can reduce the risk of developing depressive symptoms and improve the quality of life among institutionalised people. In addition, internal and external social support plays a crucial role in predicting the quality of life of institutionalised older adults, with a strong association found between social support and quality of life, i.e., social support can be considered a predictor of quality of life in institutionalised older adults [41].

Therefore, understanding the association between depressive symptomatology, negative feelings of loneliness, and perceived quality of life in institutionalised versus non-institutionalised older adults seems important so that we can find strategies to intervene, minimising the consequences of depressive symptomatology and consequently providing a better perception of negative feelings of loneliness and overall quality of life.

We found no recent studies investigating the association between the prevalence of depressive symptoms in institutionalised and non-institutionalised older adults and the association between these depressive symptoms, negative feelings of loneliness and perceived quality of life in Portugal in the aftermath of the COVID-19 pandemic.

Based on the literature review, we formulated the following hypothesis:

**Hypothesis 1 (H1).**  *There is a significant correlation between depressive symptomatology in institutionalised and non-institutionalised older adults and their levels of negative feelings of loneliness and perceived quality of life.*

Thus, our study aims to estimate the prevalence of depressive symptomatology among older adults and to assess their association with loneliness and quality of life according to institutionalisation status in a Portuguese sample.

## 2. Materials and Methods

### 2.1. Sample

#### 2.1.1. Inclusion and Exclusion Criteria

Inclusion criteria: age 60 or over, with the capacity to give informed consent or, alternatively, informed consent given by a legal representative and sufficient command of the Portuguese language. Exclusion criteria: people who refused to take part in the study and individuals with serious psychiatric disorders such as schizophrenia and bipolar disorder, as well as participants with cognitive impairment, to reduce study bias.

#### 2.1.2. Participant Selection Process

A total of 1522 participants were initially contacted who were users of Portuguese institutions, including nursing homes and residential facilities, both institutionalised and non-institutionalised (day centres, where older adults go to dinner and sleep at home). Participants were selected by convenience sampling and were recruited from institutions that agreed to take part in the study in Portugal. Of the 1522 participants contacted, 1512 were aged 60 or over, 980 met the exclusion criteria, and 7 could not be given the PHQ-9 instrument (due to participants giving up on taking part in the study, and so it was not possible to harvest this instrument). The study lasted four months, from September to December 2023. The participants filled in the instruments in the presence of the researcher. Data from 525 participants was analysed.

**Figure 1:**
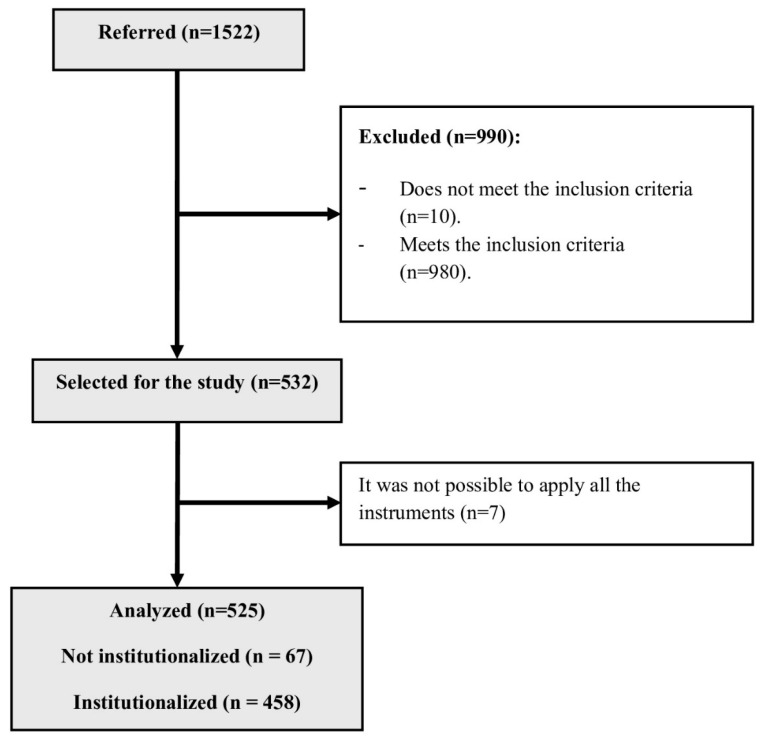
Flowchart showing the flow of participants during the study.

After all the participants had given their informed consent, all the instruments were applied to the participants. All participants who agreed to take part in the study were given a code to guarantee their anonymity. Figure 1 shows the flow chart of participants during the study.

#### 2.1.3. Ethical Considerations

Participation in this study and its objectives were explained to the participants, and informed consent was obtained. This study was approved by the ethics committee of the University of Évora—opinion No. 22073. The confidentiality of the participants was safeguarded in all the study procedures. All procedures took into account the 1964 Declaration of Helsinki and its subsequent amendments [42].

### 2.2. Measures

#### 2.2.1. Sociodemographic Questionnaire—From the Elderly Nursing Core Set (ENCS)

We used the sociodemographic questionnaire from the Elderly Nursing Core Set (ENCS) [43] in which we questioned, for example, age, sex, marital status, level of education, associated illnesses, whether they were visited by family or friends, and whether they spent any time with family and friends because we considered it appropriate to the objectives of this study.

#### 2.2.2. Patient Health Questionnaire (PHQ-9)

The PHQ-9 was developed by Kroenke et al., 2001, with the aim of assessing the presence of depressive symptoms. The PHQ-9 was adapted for the Portuguese population by Monteiro et al. (2013) and has satisfactory internal consistency (Cronbach’s alpha = 0.86) and strong convergent validity with the BDI (r = 0.85; *p* < 0.01) [44]. This instrument consists of nine items that assess the severity of symptoms associated with depression on a 4-point Likert scale (from 0 “Never” to 3 “Almost every day”). The summed scores range from 0 to 27. Scores between 0 and 4 indicate minimal depressive symptomatology, scores between 5 and 9 indicate mild depressive symptomatology, scores between 10 and 14 indicate moderate depressive symptomatology, scores between 15 and 19 indicate moderately severe depressive symptomatology and scores between 20 and 27 indicate severe depressive symptomatology [45]. In the final model, we compared mild, moderate, moderately severe or severe symptoms with no or minimal symptoms.

#### 2.2.3. WHOQOL-BREF

This instrument is a shortened version of the original WHOQOL-100 instrument, which consists of 100 questions that assess six domains: physical, psychological, level of independence, social relationships, environment and spirituality/beliefs and personal/religiosity. The WHOQOL-BREF consists of two general questions about the perceived general quality of life and satisfaction with health and 26 items that assess four domains: physical, social relationships, environment and psychological [32]. The WHOQOL-BREF (World Health Organization Quality of Life Instruments—Brief) was developed by the World Health Organization in 1998 [46] and was adapted for the Portuguese population by Vaz Serra et al. (2006) The psychometric characteristics of the instrument have shown good internal consistency [32,47].

#### 2.2.4. Loneliness Scale—UCLA

UCLA, currently in its 3rd version developed by Russell (1996), was adapted for the Portuguese elderly population by Pocinho, Farate and Dias (2010) and is used to assess negative feelings of loneliness. The study revealed temporal stability [α (16 items) = 0.985], high internal consistency (Cronbach’s alpha 0.905) and factorial validity (2 factors), which showed that the UCLA scale has high reliability as an instrument for diagnosing loneliness in older adults. The UCLA consists of 16 items, with two dimensions (social isolation and affinities), each item having four response options: “Often”, “Sometimes”, “Rarely” and “Never”. If the overall score is >32, it indicates negative feelings of loneliness (the higher the score, the greater the feeling of loneliness). It is an instrument of hetero-administration [48,49].

## 3. Statistical Analysis

Statistical analysis was performed using STATA 15.1 [50]. Data were described as counts and proportions for categorical variables and means and standard deviations (SDs) for normally distributed continuous variables. Unconditional logistic regression models were fitted to compute crude adjusted odds ratios (ORs) and the respective 95% confidence intervals (95%CIs) for the association between sociodemographic, clinical and psychosocial characteristics and depressive symptomatology, according to institutionalisation status. The final model was adjusted for sex, age, QoL and feelings of loneliness.

## 4. Results

In the present study, 69.3% of the participants were female, 81.5% were aged 80 years old or older, 17.3% were married or cohabiting, 33% did not attend school, and the majority (95.3%) reported the diagnosis of 2 or more chronic diseases (Table 1). The mean (SD) QoL ranged from 52.8 (10.9) for the physical dimension to 63.6 (13.3) for the environmental dimension, with a mean value (SD) of overall QoL equal to 53.4 (18.7). More than a third (34.2%) of the older adults assessed presented negative feelings of loneliness, with a mean (SD) score of 29.2 (10.2) on the UCLA-16 scale. Women and younger participants were more frequently non-institutionalised (*p* = 0.015 and *p* < 0.001, respectively), while those institutionalised reported more frequent feelings of loneliness (*p* = 0.047). There were no significant differences between non-institutionalised and institutionalised older adults regarding marital status, educational level, multimorbidity and the perception of QoL for any of the evaluated domains.

**Figure 2:**
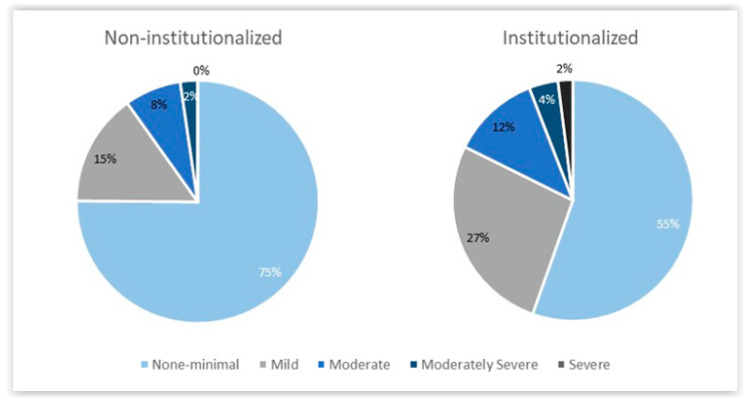
Prevalence of depressive symptoms assessed by the Patient Health Questionnaire-9 (PHQ-9), according to institutionalisation status.

As can be seen in Figure 2, in comparison to institutionalised older adults, non-institutionalised older adults had a significantly lower mean (SD) score of depressive symptomatology on the PHQ-9 questionnaire (3.3 (4.3) and 5.2 (5.1), *p* = 0.005). Specifically, 74.6% of non-institutionalised participants had no or minimal symptoms of depression, while 55.4% of institutionalised older adults fell into this category. Mild to moderately severe depressive symptoms were present in 25.4% of non-institutionalised participants, while 26.9% of institutionalised participants had mild symptoms, 11.8% had moderate symptoms, 3.9% had moderately severe symptoms and 2.0% had severe depressive symptoms.

As can be seen in Table 2, there were no statistically significant differences between participants reporting mild, moderate, moderately severe or severe symptoms in comparison to those reporting none or minimal symptoms of depression regarding sex, age, marital status, educational level and morbidity. This was observed for both non-institutionalised and institutionalised older adults.

## 5. Discussion

In Portugal, institutionalised older adults tend to be mainly women with multimorbidity [51], which is in line with our study because women in Portugal have a longer life expectancy than men, and due to age, multimorbidity is common. Overall, institutionalised older adults are more likely to be single [52] because if they are married, they may have some support from their spouse, and with an average age of 84 [51] because at older ages, they are more likely to need the support of institutionalisation, which is also in line with our study.

Our results showed that non-institutionalised older adults had a significantly lower mean score for depressive symptomatology compared with institutionalised older adults, and 74.6% of non-institutionalised participants had no or minimal depressive symptoms, while 55.4% of institutionalised participants fell into this category. These results are in line with previous studies reporting that institutionalised older adults tend to have higher rates of depressive symptoms compared with non-institutionalised older adults [23], and in addition, older adults living in nursing homes had higher levels of depressive symptomatology compared with those living in extended households [25].

In our study, 44.6% of institutionalised older adults had some kind of depressive symptomatology (26.9% of the participants had mild depressive symptoms, 11.8% had moderate depressive symptoms, 3.9% had moderately severe depressive symptoms and 2.0% had severe depressive symptoms), and “only” 25.4% of non-institutionalised older adults had some kind of depressive symptomatology (15% of the participants had mild depressive symptoms, 8% had moderate depressive symptoms, 2% had moderately severe depressive symptoms and 0% had severe depressive symptoms). In fact, institutionalisation and its association with depressive symptoms have been studied, and our results are corroborated by previous studies [40,53]. This may be due to the fact that institutionalisation can lead to a decrease in the frequency with which older adults socialise with friends and family; it can lead to a change of place of residence with a whole new set of routines that alter the daily life that the older adult was used to, and it can lead to moving away from the city they have lived in for many years and less participation in leisure activities.

Although the results show a greater volume of depressive symptoms (and more severe ones) in institutionalised older adults, these results highlight the importance of developing interventions aimed at minimising depressive symptoms in institutionalised older adults, but also in non-institutionalised older adults. This is because, although non-institutionalised older adults had depressive symptoms of around 25.4% (lower than institutionalised participants), this seems to indicate that the COVID-19 pandemic had increased depressive symptoms in older adults overall because before the pandemic, it was estimated that 5.7 per cent of older adults had depressive symptoms [17,54], and this percentage increased to 28.1% due to the COVID-19 pandemic [18]. In Portugal, the pandemic was responsible for 52.9 per cent of older adults having depressive symptoms [55]. These figures were maintained overall in our study despite the pandemic crisis having ended.

In fact, during the COVID-19 pandemic, the prevalence of depressive symptoms in older adults, both institutionalised and non-institutionalised, has increased, with institutionalised people suffering the greatest impact [56].

Our study shows that the prevalence of depressive symptoms in older adults has not decreased to pre-pandemic levels. We know that the COVID-19 pandemic has increased the prevalence of depressive symptomatology, and our study shows that this prevalence remains overall in the older adults studied, even though we might have expected it to fall once the pandemic crisis was over. According to the results of our study, the prevalence of depressive symptoms continues in this population, which may come as a surprise because we are no longer in a serious pandemic crisis.

Although there is no longer an active pandemic crisis, one study reports that the prevalence of depressive symptoms in older adults has increased during the COVID-19 pandemic, with rates of 18.9 per cent, 28.1 per cent and 35.9 per cent before, during and 10 months after the outbreak [57] and, according to our study, there is still a fairly significant prevalence of depressive symptoms in this population today. In addition, older adults with a history of depressive symptoms are at greater risk of worsening symptoms during crises such as the COVID-19 pandemic [58], emphasising the importance of intervening in this population in order to be better prepared for a similar crisis in the future.

That is, the very significant pandemic consequences in terms of the increase in depressive symptoms in older adults [59] continue today, indicating that interventions are needed to minimise depressive symptoms in institutionalised and non-institutionalised older adults despite the greater severity in institutionalised participants.

These findings emphasise the urgent need to develop interventions aimed at older adults to address the increase in the prevalence of depressive symptoms and their maintenance at high levels among older adults, especially in the post-pandemic period that we are experiencing, contrary to what we might suppose.

In our study, institutionalised participants reported negative feelings of loneliness more often, which is in line with previous studies that report that although negative feelings of loneliness are present in both institutionalised and non-institutionalised older adults, the situation is more acute in institutionalised ones [60,61].

Several studies have reported that negative feelings of loneliness are associated with the development of depressive symptoms [62,63,64], including a review with meta-analysis that reports that loneliness is an important variable in the onset of depressive symptoms [28], which is in line with our study in which participants who described negative feelings of loneliness were also more likely to have depressive symptoms (OR = 78.10; 95%CI 2.90–2106.08 and OR = 3.53; 95%CI 1.72–6.91 for non-institutionalised and institutionalised older adults, respectively), regardless of the institutionalisation situation.

Before the COVID-19 pandemic, the negative feeling of loneliness was already considered a risk factor for depressive symptoms [63,64], which has continued during the COVID-19 pandemic [65] understandably due to the containment measures implemented to contain the SARS-CoV-2 virus, and according to our study, feelings of loneliness remain an important variable to consider in older adults in the context of mental health.

A recent Scoping Review reports that in the pre-COVID-19 era, estimates of feelings of loneliness were highest in Nigeria (46 per cent) and lowest in Australia (5 per cent), with an average prevalence of 25.6 per cent. It was also reported that the prevalence of feelings of loneliness was higher in institutionalised older adults than in the general population, with an estimated average prevalence of 47.8%, adding that during the pandemic crisis, the prevalence of feelings of loneliness was higher than before the pandemic, with an average prevalence of 39.4%, compared with 25.6 before the pandemic [66].

In fact, a substantial proportion of institutionalised older adults report moderate to severe loneliness [60], emphasising the importance of addressing this problem. And, in this sense, it seems important to develop proactive interventions to minimise negative feelings of loneliness in older adults and thus, by minimising negative feelings of loneliness, also intervene in depressive symptomatology, and our results corroborate this need.

It might have been expected, once the lockdown measures to contain the SARS-CoV-2 virus had ended, that the negative feelings of loneliness reported would be fewer in number. However, this did not happen, so we think it is important to develop interventions that promote social interaction and thus minimise negative feelings of loneliness. In fact, these interventions seem to play an important role in the perceived quality of life of older adults. Studies suggest that approaches that facilitate and promote social interaction between institutionalised and non-institutionalised older adults significantly improve their quality of life (Li et al., 2023) and, consequently, their mental health [67].

In our study, despite the lack of statistical evidence for some domains, a higher quality of life was associated with lower levels of depressive symptoms. After adjustment for sex, age, QoL and feelings of loneliness, the physical domain (OR = 0.94; 95%CI 0.91–0.98) and the general perception of QoL (OR = 0.96; 95%CI 0.95–0.98) remained inversely associated with having mild to severe symptoms of depression, among older adults living in institutions. This is in line with previous studies in which depressive symptomatology is inversely associated with the perception of quality of life [68,69].

We know that in many cases, institutionalised and non-institutionalised older adults do not have any activities or do so in small numbers due to various factors. We also know that restricting activities significantly reduces health-related quality of life and increases the likelihood of developing depressive symptoms [68]. Our study suggests that a lower perception of quality of life is associated with the development of depressive symptoms, and in this sense, it is necessary to develop interventions that can contribute to a higher perception of quality of life, which may involve planning activities and thus contribute to a reduction in depressive symptoms.

It is also important to emphasise that intervening in depressive symptoms is crucial to improving quality of life and, of course, general mental well-being [70,71], which implies a holistic approach to nursing care for older adults. A review of the literature emphasises that care centred on the person with depressive symptoms must take into account shared decision-making and respond to the specific needs of the person, taking their preferences into account [72].

However, the universe of effective interventions for reducing depressive symptoms in older adults extends to virtual interventions, such as cognitive-behavioural therapy via the internet or telephone, which have also shown promise in reducing depressive symptoms, as reported in a review by Goodarzi et al. (2023) Remote interventions (via the internet or telephone) have already been reported to be effective in reducing depressive symptoms in older adults during the pandemic period [73,74,75,76]. This leads us to propose that different intervention methodologies are effective in reducing depressive symptoms in older adults.

Ageing compromises the physical and psychological capacities of older adults. Nurses who practice in geriatric populations play a crucial role in promoting the mental health of this population. The implementation of nursing strategies that focus on the perceived quality of life of institutionalised older adults has been effective in improving their perceived quality of life [77,78]. On the other hand, it is also important for nurses who practice with older adults to have education and training on the psychological consequences associated with ageing so that they are better able to intervene [78], and they can also be vehicles for change among the carers of older adults through specific training for them [79].

We therefore believe that nurses should intervene directly with older adults, but this role is much broader, as they can implement training programmes with their careers.

Essentially, we know that the COVID-19 pandemic has increased the prevalence of depressive symptoms, and currently, these figures have not decreased to pre-pandemic values, which emphasises the importance of nurses developing interventions with older adults to reduce depressive symptoms and feelings of loneliness and improving perceived quality of life. It might have been expected that the prevalence of depressive symptoms would have decreased compared with the pandemic period and at least returned to pre-pandemic levels, but this has not happened, and we feel it is important to know these data.

The development with older adults of psychotherapeutic interventions such as psychotherapy, cognitive-behavioural therapy, behavioural therapy and reminiscence therapy can be very useful in reducing depressive symptoms in older adults living in different environments, including those living in long-term care institutions [80,81]. Research into interpersonal psychotherapy also emphasises its effectiveness in reducing depressive symptoms and preventing relapses in depressed patients and also shows its effectiveness as a good intervention option for older adults with depressive symptoms, as reported in a recent Randomised Control Trial [82].

To summarise, although we are no longer in the presence of a pandemic crisis that has led to an increase in the prevalence of depressive symptoms in older adults, this prevalence has not decreased to pre-pandemic levels, nor have the negative feelings of loneliness reported, which consequently reduces the perceived quality of life. This result of “no decrease” cannot be ignored and seems relevant to us. This finding is important because it emphasises the great need for nurses who practice with older adults to develop interventions aimed precisely at reducing depressive symptoms, reducing negative feelings of loneliness and improving perceived quality of life. Nurses can play a decisive role in this objective.

How the provision of quality nursing care adapted to the needs of older adults can have a positive impact on outcomes and individual satisfaction, we recommend that nurses focus on interventions aimed at reducing depressive symptoms, reducing negative feelings of loneliness and improving perceived quality of life, in order to promote the mental health of older adults. Creating a compassionate environment and facilitating interaction between older adults and family and friends will also be important.

These results are relevant to the competencies of nurses specialising in mental health nursing, as they can provide quality and safe care centred on the person and the family, including risk assessment and management, understanding the principles of recovery, communication skills, knowledge about mental disorders and their treatment, research evaluation and the promotion of physical and mental health. And, in this sense, they can develop a range of interventions with older adults in pursuit of these competencies; they are trained to do so.

### Limitations

This study has some limitations. Firstly, 69.3% of the participants were female, which is a high percentage compared with males; a total of 80.5% were aged 80 or over in a sample with participants aged 60 or over, which does not allow us to draw conclusions at ages closer to 60; and a very different number between institutionalised and non-institutionalised older adults.

## 6. Conclusions

The results of this study are relevant to clinical practice, teaching and research. Firstly, in terms of clinical practice, the results of this study could lead to an improvement in the quality and safety of care provided to older adults, as it highlights the need to intervene with the aim of reducing depressive symptoms, reducing negative feelings of loneliness and improving quality of life. In terms of teaching, the results of this study can be used to increase their analysis and discussion to promote students’ critical thinking and thus prepare them to make informed decisions based on scientific evidence. In terms of research, the results of this study could lead to the development of new approaches to advance scientific knowledge.

The prevalence of depressive symptomatology is high in institutionalised and non-institutionalised older adults, which means that it has not fallen following the increase due to the COVID-19 pandemic, contrary to what might have been expected. A lower perception of quality of life and the presence of negative feelings of loneliness are associated with the onset of depressive symptoms. These conclusions suggest that nursing intervention plans should be developed to intervene in the dimensions of depressive symptomatology, perceived quality of life and negative feelings of loneliness.

These interventions should address mental health challenges, in particular depressive symptoms, but they can also make a decisive contribution to reducing negative feelings of loneliness, thus contributing to a more positive perception of quality of life among older adults. More important are these intervention plans so that we are better prepared for a future pandemic situation.

We recommend similar studies in the future with a more equal sample of male and female participants and a more balanced age distribution.

## Figures and Tables

**Table 1:** Participant characteristics according to institutionalisation status.

	Overall (*n* = 525)	Non-Institutionalised (*n* = 67)	Institutionalised (*n* = 458)	*p*
Sex, *n* (%)				
Male	161 (30.7)	12 (17.9)	149 (32.5)	
Female	364 (69.3)	55 (82.1)	309 (67.5)	0.015
Age (years), *n* (%)				
60–79	97 (18.5)	25 (31.3)	72 (15.7)	
≥80	428 (81.5)	42 (62.7)	389 (84.3)	<0.001
Marital status, *n* (%)				
Married/cohabiting	91 (17.3)	14 (20.9)	77 (16.8)	
Single/widowed/divorced/separated	434 (82.7)	53 (79.1)	381 (83.2)	0.410
Educational level, *n* (%)				
Did not attend school	173 (33.0)	28 (41.8)	145 (31.7)	
Attended school	352 (67.0)	39 (58.2)	313 (68.3)	0.099
Multimorbidity ^¥^, *n* (%)				
No	25 (4.8)	5 (7.5)	20 (4.4)	
Yes	500 (95.3)	62 (92.5)	438 (95.6)	0.266
Quality of life (WHOQOL-BREF) *, mean (SD)				
Physical	52.8 (10.9)	52.9 (9.6)	52.8 (11.1)	0.913
Psychological	55.0 (13.1)	57.8 (13.5)	54.6 (13.0)	0.062
Social Relationships	60.8 (13.6)	63.4 (12.7)	60.4 (13.7)	0.102
Environment	63.6 (13.3)	64.6 (11.3)	63.4 (13.6)	0.503
Overall	53.4 (18.7)	57.8 (16.0)	52.5 (19.1)	0.061
Feelings of loneliness (UCLA-16) ^¶^, *n* (%)				
No	317 (65.8)	47 (77.1)	270 (64.1)	
Yes	165 (34.2)	14 (22.9)	151 (35.9)	0.047

^¥^ Diagnosis of 2 or more chronic diseases. * World Health Organization Quality of Life Instruments —Brief (range: 0–100). ^¶^ UCLA—Loneliness Scale.

**Table 2:** Crude and adjusted association between participants’ sociodemographic, clinical and psychosocial characteristics and depressive symptomatology, according to institutionalisation status.

	Non-Institutionalised		Institutionalised	
	Crude OR (95%CI)	Adjusted OR (95%CI) ^1^	Crude OR (95%CI)	Adjusted OR (95%CI) ^1^
Sex				
Male	1	1	1	1
Female	1.02 (0.24–4.33)	0.20 (0.01–3.69)	1.19 (0.80–1.77)	1.21 (0.61–2.39)
Age (years)				
60–79	1	1	1	1
≥80	0.58 (0.19–1.77)	0.07 (0.01–1.12)	1.32 (0.79–2.20)	2.35 (0.94–5.83)
Marital status				
Married/cohabiting	1	-	1	-
Single/widowed/divorced/separated	0.53 (0.15–1.87)	-	1.02 (0.62–1.67)	-
Educational level				
Did not attend school	1	-	1	-
Attended school	1.03 (0.34–3.16)	-	1.11 (0.75–1.65)	-
Multimorbidity ^¥^				
No	1	-	1	-
Yes	1.39 (0.14–13.39)	-	0.64 (0.26–1.59)	-
Quality of life (WHOQOL-BREF) *				
Physical	0.87 (0.80–0.94)	0.80 (0.63–1.02)	0.92 (0.90–0.94)	0.94 (0.91–0.98)
Psychological	0.96 (0.92–1.00)	0.97 (0.86–1.10)	0.95 (0.94–0.97)	0.99 (0.96–1.03)
Social Relationships	0.97 (0.92–1.01)	1.08 (0.96–1.22)	0.97 (0.95–0.98)	0.99 (0.97–1.03)
Environment	0.95 (0.90–1.01)	0.97 (0.85–1.10)	0.96 (0.95–0.98)	1.02 (0.98–1.05)
Overall	0.93 (0.87–0.99)	1.03 (0.91–1.17)	0.95 (0.93–0.96)	0.96 (0.95–0.98)
Feelings of loneliness (UCLA-16) ^¶^				
No	1	1	1	1
Yes	10.29 (2.64–39.93)	78.10 (2.90–2106.08)	5.93 (3.83–9.19)	3.53 (1.72–6.91)

^1^ Adjusted for sex, age, QoL and feelings of loneliness. ^¥^ Diagnosis of 2 or more chronic diseases. * World Health Organization Quality of Life Instruments—Brief. ^¶^ UCLA—Loneliness Scale.

## Data Availability

The data presented in this study are available upon request from the corresponding author.

## Public Involvement Statement

No public involvement in any aspect of this.

## Guidelines and Standards Statement

This manuscript was drafted against the Strengthening the Reporting of Observational Studies in Epidemiology (STROBE) for cross-sectional studies.

## References

[1] Zafar J., Malik N.I., Atta M., Makhdoom I.F., Ullah I., Manzar D. (2021). Loneliness may mediate the relationship between depression and the quality of life among elderly with mild cognitive impairment. Psychogeriatrics.

[2] Kraav S.-L., Lehto S.M., Junttila N., Ruusunen A., Kauhanen J., Hantunen S., Tolmunen T. (2021). Depression and loneliness may have a direct connection without mediating factors. Nord. J. Psychiatry.

[3] Nascimento M.d.M., Lampraki C., Marques A., Gouveia É.R., Adsuar J.C., Ihle A. (2024). Longitudinal cross-lagged analysis of depression, loneliness, and quality of life in 12 European countries. BMC Public Health.

[4] Inoue K., Haseda M., Shiba K., Tsuji T., Kondo K., Kondo N. (2023). Social Isolation and Depressive Symptoms Among Older Adults: A Multiple Bias Analysis Using a Longitudinal Study in Japan. Ann. Epidemiol..

[5] Chang C.H., Lee K.-Y., Shim Y.H. (2017). Normal aging: Definition and physiologic changes. J. Korean Med. Assoc..

[6] Strulik H. (2023). The Routledge Handbook of the Economics of Ageing.

[7] Amarya S., Singh K., Sabharwal M. (2018). Ageing Process and Physiological Changes. Gerontology.

[8] Schmauck-Medina T., Molière A., Lautrup S., Zhang J., Chlopicki S., Madsen H.B., Cao S., Soendenbroe C., Mansell E., Vestergaard M.B. (2022). New hallmarks of ageing: A 2022 Copenhagen ageing meeting summary. Aging.

[9] United Nations (2015). World Population Ageing [Highlights] [Internet]. Department of Economic and Social Affairs. https://www.un.org/en/development/desa/population/publications/pdf/ageing/WPA2015_Highlights.pdf.

[10] Armitage R., Nellums L.B. (2020). COVID-19 and the consequences of isolating the elderly. Lancet Public Health.

[11] Newman M.G., Zainal N.H. (2020). The value of maintaining social connections for mental health in older people. Lancet Public Health.

[12] Correia T.S.P., Martins M.M.F.P.S., Barroso F.F., Pinho L.G., Fonseca C., Valentim O., Lopes M. (2022). The Implications of Family Members’ Absence from Hospital Visits during the COVID-19 Pandemic: Nurses’ Perceptions. Int. J. Environ. Res. Public Health.

[13] World Health Organization (2015). World Report on Ageing and Health.

[14] Ahadi B., Hassani B. (2021). Loneliness and Quality of Life in Older Adults: The Mediating Role of Depression. Ageing Int..

[15] Arco H., Pedro A., Pinho L., Proença A. (2021). Aging and Functionality of the Institutionalized Elderly People of Alto Alentejo: Contributions to the Diagnosis of the Situation. International Workshop on Gerontechnology.

[16] American Psychiatric Association (2014). DSM-5 Manual de Diagnóstico e Estatística das Perturbações Mentais.

[17] James S.L., Abate D., Abate K.H., Abay S.M., Abbafati C., Abbasi N., Abbastabar H., Abd-Allah F., Abdela J., Abdelalim A. (2018). Global, regional, and national incidence, prevalence, and years lived with disability for 354 diseases and injuries for 195 countries and territories, 1990–2017: A systematic analysis for the Global Burden of Disease Study 2017. Lancet.

[18] Santomauro D.F., Herrera A.M.M., Shadid J., Zheng P., Ashbaugh C., Pigott D.M., Abbafati C., Adolph C., Amlag J.O., Aravkin A.Y. (2021). Global prevalence and burden of depressive and anxiety disorders in 204 countries and territories in 2020 due to the COVID-19 pandemic. Lancet.

[19] Moritz S., Lysaker P.H., Hofmann S.G., Hautzinger M. (2018). Going meta on metacognitive interventions. Expert Rev. Neurother..

[20] Wu J.-J., Wang H.-X., Yao W., Yan Z., Pei J.-J. (2020). Late-life depression and the risk of dementia in 14 countries: A 10-year follow-up study from the Survey of Health, Ageing and Retirement in Europe. J. Affect. Disord..

[21] Shah A., Bhat R., Zarate-Escudero S., DeLeo D., Erlangsen A. (2016). Suicide rates in five-year age-bands after the age of 60 years: The international landscape^†^. Aging Ment. Health.

[22] McDougall F.A., Matthews F.E., Kvaal K., Dewey M.E., Brayne C. (2007). Prevalence and symptomatology of depression in older people living in institutions in England and Wales. Age Ageing.

[23] Felício L.F.F., Leão L.L., e Souza E.H.E., Machado F.S.M., Laks J., Deslandes A.C., de Paula A.M.B., Monteiro-Junior R.S. (2022). Cognitive abilities of institutionalized older persons with depressive symptoms. J. Bras. Psiquiatr..

[24] Araújo Vale B., Tavares de Araújo H., Ferreira de Sena R.C., Batista Costa P., Nunes de Miranda F.A. (2023). Ideação suicida e risco de depressão entre idosos residentes em instituições de longa permanência. Rev. Baiana Enferm..

[25] Gupta A.A., Diwan S. (2023). Comparison of Depression and Quality of Life of Elderly Living in Two Alternative Residential Settings. Int. J. Health Sci. Res..

[26] Cacioppo J.T., Hawkley L.C. (2009). Perceived social isolation and cognition. Trends Cogn. Sci..

[27] Gould C.E., Carlson C., Alfaro A.J., Chick C.F., Bruce M.L., Forman-Hoffman V.L. (2021). Changes in Quality of Life and Loneliness Among Middle-Aged and Older Adults Participating in Therapist-Guided Digital Mental Health Intervention. Front. Public Health.

[28] Erzen E., Çikrikci Ö. (2018). The effect of loneliness on depression: A meta-analysis. Int. J. Soc. Psychiatry.

[29] Cacioppo S., Grippo A.J., London S., Goossens L., Cacioppo J.T. (2015). Loneliness: Clinical import and interventions. Perspect. Psychol. Sci..

[30] Domènech-Abella J., Lara E., Rubio-Valera M., Olaya B., Moneta M.V., Rico-Uribe L.A., Ayuso-Mateos J.L., Mundó J., Haro J.M. (2017). Loneliness and depression in the elderly: The role of social network. Soc. Psychiatry Psychiatr. Epidemiol..

[31] Tsai H.-H., Cheng C.-Y., Shieh W.-Y., Chang Y.-C. (2020). Effects of a smartphone-based videoconferencing program for older nursing home residents on depression, loneliness, and quality of life: A quasi-experimental study. BMC Geriatr..

[32] Vaz Serra A., Canavarro M.C., Simões M., Pereira M., Gameiro S., Quartilho M.J., Rijo D., Carona C., Paredes T. (2006). Estudos psicométricos do instrumento de avaliação da qualidade de vida da Organização Mundial de Saúde (WHOQOL-Bref) para Português de Portugal. Psiquiatr. Clínica.

[33] Cacioppo J.T., Hughes M.E., Waite L.J., Hawkley L.C., Thisted R.A. (2006). Loneliness as a specific risk factor for depressive symptoms: Cross-sectional and longitudinal analyses. Psychol. Aging.

[34] Kang H.-W., Park M., Hernandez J.P.W. (2016). The impact of perceived social support, loneliness, and physical activity on quality of life in South Korean older adults. J. Sport Health Sci..

[35] Pascut S., Feruglio S., Crescentini C., Matiz A. (2022). Predictive Factors of Anxiety, Depression, and Health-Related Quality of Life in Community-Dwelling and Institutionalized Elderly during the COVID-19 Pandemic. Int. J. Environ. Res. Public Health.

[36] Jabeur M., Gassab L., Hamdane F., Amemou B., Zaafrane F., Gaha L. (2022). Depression and quality of life in Tunisian institutionalized elderly subjects. Eur. Psychiatry.

[37] Sánchez-Anguita Muñoz Á. (2019). Depresión, socialización y autonomía en ancianos institucionalizados. Int. J. Dev. Educ. Psychol. Rev. INFAD Psicol..

[38] Mayer R.C.F., Alves M.R., Yamauti S.M., Silva M.T., Lopes L.C. (2021). Quality of Life and Functioning of People With Mental Disorders Who Underwent Deinstitutionalization Using Assisted Living Facilities: A Cross-Sectional Study. Front. Psychol..

[39] Shrestha K., Ojha S.P., Dhungana S., Shrestha S. (2020). Depression and its association with quality of life among elderly: An elderly home-cross sectional study. Neurol. Psychiatry Brain Res..

[40] Syukrowardi D.A., Suwardiman D., Kuntarto B. (2022). Social Support Improves the Quality of Life Among Institutionalized Older People in The Social Protection Center, Banten Province, Indonesia. J. Health Sci. Nurs. Stud..

[41] World Medical Association (2013). World Medical Association Declaration of Helsinki: Ethical principles for medical research involving human subjects. JAMA.

[42] Fonseca C., Lopes M., Mendes D., Parreira P., Mónico L., Marques C. (2019). Psychometric Properties of the Elderly Nursing Core Set. Gerontechnology: First International Workshop, IWoG 2018, Cáceres, Spain, and Évora, Portugal, 14 and 17 December, 2018, Revised Selected Papers 1.

[43] Kroenke K., Spitzer R.L., Williams J.B.W. (2001). The PHQ-9. J. Gen. Intern. Med..

[44] Monteiro S., Torres A., Pereira A., Albuquerque E., Morgadinho R. (2013). 2077—Preliminary validation study of a portuguese version of the patient health questionnaire (PHQ-9). Eur. Psychiatry.

[45] The Whoqol Group (1998). The World Health Organization quality of life assessment (WHOQOL): Development and general psychometric properties. Soc. Sci. Med..

[46] Vilar M., Simões M.R., Sousa L.B., Firmino H., Paredes T., Lima M.P., Canavarro M.C., Serra A.V. (2010). Qualidade de vida e saúde: Uma abordagem na perspectiva da Organização Mundial de Saúde. WHOQOL Disponível para Portugal: Desenvolvimento dos Instrumentos de Avaliação da Qualidade de Vida da Organização Mundial de Saúde (WHOQOL-100 e WHOQOL-BREF).

[47] Russell D. (1996). UCLA Loneliness Scale (Version 3): Reliability, Validity, and Factor Structure. J. Pers. Assess..

[48] Pocinho M., Farate C., Dias C.A. (2010). Validação Psicométrica da Escala UCLA-Loneliness para Idosos Portugueses. Interações Soc. Novas Mod..

[49] College Station T (2017). Stata Statistical Software: Release 15.1 College Station, TX, 2017 [Internet]. https://www.stata.com/features/documentation/.

[50] Ferreira C.R., Mascarenhas-Melo F., Rodrigues A.R., Lima M.J.R., Pinheiro J.P., Chaves C., Teixeira-Lemos E., Bell V. (2022). Characterisation of institutionalised Portuguese older adult fallers: Is there a place for pharmacist intervention? A preliminary study. Pharm. Pract..

[51] Benksim A., Addi R.A., Khalloufi E., Habibi A., Cherkaoui M. (2021). Self-reported morbidities, nutritional characteristics, and associated factors in institutionalized and non-institutionalized older adults. BMC Geriatr..

[52] Rebouças C.M.d.P., Ribeiro M.R., Zangilorami-Raimundo J., Bezerra P.C.d.L., Júnior A.M.d.C.d.S., Souza N.d.S., Pereira J.R., Júnior J.M.S., Costa L.M.d.P.R.d., de Abreu L.C. (2021). Additional file 1 of Association between sleep quality and depression among institutionalized and community older people—Brazilian Western Amazonia. BMC Psychiatry.

[53] Organisation W.H. (2021). Depression. https://www.who.int/news-room/fact-sheets/detail/depression.

[54] Carvalho J., Borges-Machado F., Pizarro A.N., Bohn L., Barros D. (2021). Home Confinement in Previously Active Older Adults: A Cross-Sectional Analysis of Physical Fitness and Physical Activity Behavior and Their Relationship With Depressive Symptoms. Front. Psychol..

[55] Pavlidou E., Papadopoulou S.K., Antasouras G., Spanoudaki M., Mentzelou M., Dimoliani S., Tsourouflis G., Psara E., Vorvolakos T., Dakanalis A. (2024). Evaluating the sociodemographic, anthropometric and lifestyle parameters, depression, quality of life, cognitive status, physical activity, and Mediterranean diet adherence of older adults in pre- and post-Covid-19 periods: A comparative cross-sectional study. Psychol. Health.

[56] Wang Y., Luo B., Wang J., Liao S. (2023). The psychological impact of the COVID-19 pandemic in the elderly in southwest China: A longitudinal study based on generalized estimating equations. Int. J. Disaster Risk Reduct..

[57] Zhu J., Zaninotto P., Di Gessa G. (2023). Pre-pandemic trajectories of depressive symptomatology and their relation to depression during the COVID-19 pandemic: Longitudinal study of English older people. BJPsych Open.

[58] Silva C., Fonseca C., Ferreira R., Weidner A., Morgado B., Lopes M.J., Moritz S., Jelinek L., Schneider B.C., Pinho L.G. (2023). Depression in older adults during the COVID-19 pandemic: A systematic review. J. Am. Geriatr. Soc..

[59] Hajek A., Zwar L., Gyasi R.M., Kretzler B., König H.-H. (2023). Prevalence and determinants of loneliness among the oldest old living in institutionalized settings. Z. Gerontol. Geriatr..

[60] Just S.A., Seethaler M., Sarpeah R., Waßmuth N., Bermpohl F., Brandl E.J. (2022). Loneliness in Elderly Inpatients. Psychiatr. Q..

[61] Wang G., Hu M., Xiao S.-Y., Zhou L. (2017). Loneliness and depression among rural empty-nest elderly adults in Liuyang, China: A cross-sectional study. BMJ Open.

[62] Zhao X., Zhang D., Wu M., Yang Y., Xie H., Li Y., Jia J., Su Y. (2018). Loneliness and depression symptoms among the elderly in nursing homes: A moderated mediation model of resilience and social support. Psychiatry Res..

[63] Alibasic E., Ramic E., Bajraktarevic A., Karic E., BaticMujanovic O., Ramic I., Alibasic E. (2018). Geriatric Depression in Family Medicine. Mater. Socio-Medica.

[64] Kurniawidjaja M., Susilowati I.H., Erwandi D., Kadir A., Hasiholan B.P., Al Ghiffari R. (2022). Identification of Depression Among Elderly During COVID-19. J. Prim. Care Community Health.

[65] Schroyen S., Janssen N., Duffner L.A., Veenstra M., Pyrovolaki E., Salmon E., Adam S. (2023). Prevalence of Loneliness in Older Adults: A Scoping Review. Health Soc. Care Community.

[66] Newman-Norlund R.D., Newman-Norlund S.E., Sayers S., McLain A.C., Riccardi N., Fridriksson J. (2022). Effects of social isolation on quality of life in elderly adults. PLoS ONE.

[67] Sun W., Lu H., Huang F., Shiu C.-S., Zhang L., Chen W.-T. (2021). Longitudinal trajectory of the association between quality of life and depression among people living with HIV in China: A mixed effects model. AIDS Care.

[68] Lee D.-Y., Kim S.-G. (2020). The Association between Health-related Quality of Life and Depression on Activity Restriction in Osteoarthritis: A Cross-sectional Study. J. Korean Phys. Ther..

[69] Papageorgiou A., Bakola M., Kitsou K., Mousafeiris V., Mavridou K., Kallianezos P., Kampouraki M., Charalambous G., Jelastopulu E. (2022). The association between depression and quality of life in the elderly. Eur. J. Public Health.

[70] Shah F., Solanki P., Tandel N. (2022). A Study to Find Correlation between Depression and Quality of Life in Geriatric Population. Int. J. Sci. Health Res..

[71] Pinho L.G., Lopes M.J., Correia T., Sampaio F., Arco H.R., Mendes A., Marques M.D., Fonseca C. (2021). Patient-Centered Care for Patients with De-pression or Anxiety Disorder: An Integrative Review. J. Pers. Med..

[72] Goodarzi Z., Holroyd-Leduc J., Seitz D., Ismail Z., Kirkham J., Wu P., Fox L., Hykaway W., Grossman L., Ewa V. (2022). Efficacy of virtual interventions for reducing symptoms of depression in community-dwelling older adults: A systematic review. Int. Psychogeriatr..

[73] Gilbody S., Littlewood E., McMillan D., Chew-Graham C.A., Bailey D., Gascoyne S., Sloan C., Burke L., Coventry P., Crosland S. (2021). Behavioural activation to prevent depression and loneliness among socially isolated older people with long-term conditions: The BASIL COVID-19 pilot randomised controlled trial. PLoS Med..

[74] Pellas J., Renner F., Ji J.L., Damberg M. (2021). Telephone-based behavioral activation with mental imagery for depression: A pilot randomized clinical trial in isolated older adults during the Covid-19 pandemic. Int. J. Geriatr. Psychiatry.

[75] Shapira S., Yeshua-Katz D., Cohn-Schwartz E., Aharonson-Daniel L., Sarid O., Clarfield A.M. (2021). A pilot randomized controlled trial of a group intervention via Zoom to relieve loneliness and depressive symptoms among older persons during the COVID-19 outbreak. Internet Interv..

[76] Malathi P. (2022). Effectiveness of nursing strategies on quality of life among elderly living in selected old age homes. Int. J. Adv. Psychiatr. Nurs..

[77] Fathy A., Mourad G.M., El-Fatah W.O.A. (2020). Quality of Life among Elderly People at Geriatric Home. NILES J. Geriatr. Gerontol..

[78] Mohammed S.Z., Ahmad M.A., Farahat N.H. (2021). Nursing Intervention to Improve the Caregivers’ Practices toward Elderly Care at Geriatric Homes. Indian J. Forensic Med. Toxicol..

[79] Davison T.E., Bhar S., Wells Y., Owen P.J., You E., Doyle C., Bowe S.J., Flicker L. (2024). Psychological therapies for depression in older adults residing in long-term care settings. Cochrane Database Syst. Rev..

[80] Gellert P., Lech S., Hoppmann F., O’sullivan J.L., Kessler E.-M. (2024). Effectiveness of Psychotherapy for Community-Dwelling Vulnerable Older Adults with Depression and Care Needs: Findings from the PSY-CARE Trial. Clin. Gerontol..

[81] Varadharasu S. (2024). A Randomized Control Trial Study to Assess the Effectiveness of Interpersonal Psychotherapy on Symptom Reduction and Relapse Prevention for Depression among the Depression Patients. Indian J. Public Health.

[82] Sharkiya S.H. (2023). Impact of healthcare service quality on older people’s satisfaction at geriatric medical centres: A rapid review. J. Public Health Afr..

